# miR-96 regulates liver tumor-initiating cells expansion by targeting TP53INP1 and predicts Sorafenib resistance

**DOI:** 10.7150/jca.48333

**Published:** 2020-09-21

**Authors:** Yonggang Huang, Jin Zhang, HengYu Li, Huiping Peng, Maolin Gu, Hengjie Wang

**Affiliations:** 1 Department of Hepatic surgery, Kunshan Hospital of Traditional Chinese Medicine. Kunshan, Jiangsu Province, 215300, China.; 2 Department of Hepatic Surgery, Third Affiliated Hospital of Second Military Medical University, Shanghai, 200438, China.; 3 Department of General surgery, First Affiliated Hospital of Second Military Medical University, Shanghai, 200433, China.; 4 Department of Gastroenterology, Kunshan Hospital of Traditional Chinese Medicine. Kunshan, Jiangsu Province, 215300, China.

**Keywords:** hepatocellular carcinoma, tumor-initiating cells, miR-96, TP53INP1, sorafenib

## Abstract

Liver tumor-initiating cells (T-ICs) contribute to tumorigenesis, progression, recurrence and drug resistance of hepatocellular carcinoma (HCC). However, the underlying mechanism for the propagation of liver T-ICs remains unclear. In the present study, our finding shows that miR-96 is upregulated in liver T-ICs. Functional studies revealed that forced miR-96 promotes liver T-ICs self-renewal and tumorigenesis. Conversely, knockdown miR-96 inhibits liver T-ICs self-renewal and tumorigenesis. Mechanistically, miR-96 downregulates TP53INP1 via its mRNA 3'UTR in liver T-ICs. Furthermore, the miR-96 expression determines the responses of hepatoma cells to sorafenib treatment. Analysis of patient cohorts and patient-derived xenografts (PDXs) further demonstrate that the miR-96 may predict sorafenib benefits in HCC patients. Our findings revealed the crucial role of the miR-96 in liver T-ICs expansion and sorafenib response, rendering miR-96 as an optimal target for the prevention and intervention of HCC.

## Introduction

Hepatocellular carcinoma (HCC) is the most common liver cancer in adults and a challenging disease with poor prognosis 1, 2. The substantial heterogeneity and hierarchical organization in liver cancer support the theory of tumor initiating cells (T-ICs) or cancer stem cells (CSCs) in HCC 3, 4. T-ICs exhibit extended self-renewal potential and tumor initiating ability. Tumors that harbor an abundant T-IC population or have high expression of stemness-related genes may signal a poor clinical outcome in HCC patients 5, 6. Therefore, understanding how liver T-ICs regulate tumor initiation and progression is of key importance for future treatment strategies.

MicroRNAs (miRNAs) are a class of small non-coding RNA molecules that contain approximately 22 nucleotides 7, 8. miRNAs typically regulate post-transcriptional gene expression by interacting with sequences within the 3'-untranslational region (3'-UTR) of the target mRNA and play important roles in a variety of biological processes, including cell proliferation, differentiation, metastasis and apoptosis 9-11. Numerous studies also found that miRNAs are involved in the initiation, progression and recurrence of various tumors, including lung, liver and bladder cancer. miR-96 has been recognized as an oncogenic miRNA that is upregulated in various types of cancer. Previous studies found that miR-96 promotes metastasis of papillary thyroid cancer through targeting SDHB 12. Moreover, miR-96 also induced non-small-cell lung cancer progression through competing endogenous RNA network and affecting EGFR signaling pathway 13. However, the function of miR-96 in liver T-ICs is unknown.

In the present study, we first found that the expression of miR-96 is upregulated in liver T-ICs. Next, using loss-of-function and gain-of-function analyses in liver T-ICs, we demonstrate that miR-96 can promote the self-renewal capacity and tumorigenicity of liver T-ICs. Further mechanism study reveals that miR-96 directly target TP53INP1 in liver T-ICs. miR-96 overexpression HCC cells are resistant to sorafenib treatment. The analysis of patient cohorts and patient-derived xenografts (PDXs) demonstrated that miR-96 may predict sorafenib benefits in HCC patients. In conclusion, our findings revealed the crucial role of the miR-96 in liver T-ICs expansion and sorafenib response, rendering miR-96 an optimal target for the prevention and intervention in HCC.

## Materials and Methods

### Patients and samples

The HCC tissues were collected from surgical resections of patients without preoperative treatment at Eastern Hepatobiliary Surgery Hospital (Shanghai, China). (Inclusion criteria: The patients were diagnosed with HCC according to the AASLD diagnostic criteria for HCC; A single nodule of ≤ 5 cm or up to 3 nodules of ≤ 3 cm; absence of extrahepatic metastasis or vascular invasion; Child-Pugh class A or B without history of hepatic encephalopathy, refractory ascites, or esophageal/gastric varices bleeding; No previous anti-tumor treatment; Platelet count of > 40000 / mm3 and prothrombin time prolongation of ≤ 3 seconds; Understand the trial and endorse informed consent. Exclusion criteria: Metastatic liver cancer; Patients with heart, lung, brain, kidney dysfunction which may affect the therapeutic effect; Patients with other diseases that may affect treatments in this program; Patients with other malignancies; Pregnant and lactating women.) A group of 30 HCC specimens were used for analyzing the correlation between miR-96 and TP53INP1 mRNA expression. Three HCC specimens were used for isolating CD133 and EpCAM positive liver T-ICs. Four specimens were used for patient-derived xenograft (PDX) model. Patient informed consent was obtained and the procedure of human sample collection was approved by the Ethics Committee of Eastern Hepatobiliary Surgery Hospital.

### Sorafenib cohort

A total of 91 patients who received sorafenib for the recurrent tumors at Eastern Hepatobiliary Surgery Hospital from 2010-2016 (inclusion criteria: patients with recurrence of HCC after resection; absence of extrahepatic metastasis or vascular invasion; Child-Pugh class A or B without history of hepatic encephalopathy, refractory ascites, or esophageal/gastric varices bleeding; platelet count of > 40000/ mm^3^ and prothrombin time prolongation of ≤ 3 seconds; understand the trial and endorse informed consent. Exclusion criteria: metastatic liver cancer; patients with heart, lung, brain, kidney dysfunction which may affect the therapeutic effect; patients with other diseases that may affect treatments in this program; patients with other malignancies; pregnant and lactating women). Overall survival (OS) analysis was performed using the Kaplan-Meier method. OS was defined as the interval between the dates of recurrence and death. Detailed clinicopathological features and treatment of these patients are described in Supplementary Table 1.

### Cell lines and cell culture

Patient-derived primary HCC cultures of tumor cells were obtained from fresh tumor specimens of HCC patients as previously described. The human primary hepatoma cells were isolated by collagenase perfusion and centrifugation. Briefly, the liver cancer tissues were washed several times in pre-cooled sterile PBS buffer containing Red Blood Cell Lysis Buffer and 0.5% collagenase IV to remove blood and connective tissue; GBSS mixed enzyme solution was used for digestion. The cells were centrifuged, and the supernatant was discarded. Cell activity was detected by Trypan Blue staining, and cultured in a bottle containing complete medium heavy suspension at 37 °C and 5% CO_2_ environment culture. During this process the cell morphology was identified.

The HCC cell line HCCLM3 were purchased form the Chinese Academy of Sciences (Shanghai, China). The HCC cell line CSQT-2 was obtained from professor Shuqun Chen. The HCC cells were cultured with Dulbecco's modified Eagle's medium (DMEM) supplemented with 10% fetal bovine serum (FBS) and 2 mM L-glutamine, and 25 µg/ml of gentamicin and maintained at 37°C in 5% CO_2_ incubator. The cultured cells were digested with 0.5% trypsin and moved to a new plate twice a week. miR-96 mimic or miR-96 sponge lentivirus and their control lentivirus were purchased from Shanghai RiboBio (Guangzhou, China). The HCC cells were infected with lentivirus and then screened by puromycin as described before 14. The TP53INP1 siRNA and its control were obtained from GenePharma (Shanghai, China).

### RNA interference

Small interference RNAs (siRNAs) against TP53INP1 and NC (NC, negative control) siRNA were synthetized by Genepharma (Shanghai, China). The siRNAs were transfected into the hepatoma cells at a final concentration of 200 nM using siRNA transfection reagent according to the manufacturer's instructions (Polyplus, Illkirch, France). The cells were harvested or subjected to further downstream experiments 24-72 hours after transfection. Gene knockdown was validated by western blotting.

### Flow-cytometry analysis

For CD133^+^ and EpCAM^+^ cells sorting, primary HCC patients' cells and hepatoma cells were incubated with the primary anti-CD133 (Cat. no. 372806, Biolegend, Inc., San Diego, CA) or anti-EpCAM (Cat. no. ab8666; Abcam, USA) for 30 minutes at room temperature. The cells were then subjected to flow cytometry using a MoFlo XDP cell sorter from Beckman Coulter (Indianapolis, IN, USA) according to the manufacturer's instructions. The sorted cells from three independent experiments were subjected to Real-time PCR assay.

miR-96 mimic or miR-96 sponge and control hepatoma cells were incubated with the primary anti-EpCAM for 30 minutes at room temperature. The flow-cytometry analysis was performed using a MoFlo XDP from Beckman Coulter according to the manufacturer's instructions.

### Spheroid formation assay

miR-96 mimic or miR-96 sponge and their control hepatoma cells were cultured in a 96-well ultra-low attachment (300 cells per well) and cultured in DMEM/F12 (Gibco) media, supplemented with 1% FBS, 20 ng/mL bFGF and 20 ng/mL EGF for seven days. The total number of spheres was counted under the microscope (Olympus).

### *In vitro* limiting dilution assay

Various numbers of miR-96 mimic or miR-96 sponge and their control hepatoma cells (2, 4, 8, 16, 32, 64 cells per well, n=16) were seeded into 96-well ultra-low attachment and cultured in DMEM/F12 (Gibco) supplemented with 1% FBS, 20 ng/mL bFGF and 20 ng/mL EGF for seven days. The CSC proportions were analyzed using Poisson distribution statistics and the L-Calc Version 1.1 software program (Stem Cell Technologies, Inc., Vancouver, Canada) as previously described 15.

### *In vivo* limiting dilution assay

For the *in vivo* limiting dilution assay, miR-96 mimic or miR-96 sponge and their control hepatoma cells were mixed with Matrigel (BD) at a ratio of 1:1 and injected subcutaneously at indicated cell doses per NOD-SICD mouse (n=8). After 8 months, tumors formation was evaluated.

### Real-time PCR

For detection of mature miR-96, total RNA was subjected to reverse transcription using a TaqMan MicroRNA Reverse Transcription Kit (Applied Biosystems). qRT-PCR analysis of miR-96 expression was carried out using TaqMan MicroRNA assay kits (Applied Biosystems). Results were normalized to U6 snRNA using the comparative threshold cycle (Ct) method.

The total RNA was extracted by using Trizol reagent (Invitrogen, 15596-018). Total cDNAs were synthesized by ThermoScript TM RT-PCR system (Invitrogen, 11146-057). The total mRNA amount present in the cells was measured by RT-PCR using the ABI PRISM 7300 sequence detector (Applied Biosystems). PCR conditions included 1 cycle at 94 °C for 5 minutes, followed by up to 40 cycles of 94 °C for 15 seconds (denaturation), 60 °C for 30 seconds (annealing) and 72 °C for 30 seconds (extension). The sequences of primers used are listed in Supplementary Table 2.

### Western blotting assay

Thirty micrograms of proteins were subjected to sodium dodecyl sulfate polyacrylamide gel electrophoresis and then transferred to nitrocellulose membrane. The membrane was blocked with 5% non-fat milk and incubated with the primary antibody overnight. The protein band, specifically bound to the primary antibody, was detected using an IRDye 800CW-conjugated secondary antibody and LI-COR imaging system (LI-COR Biosciences, Lincoln, NE, USA). The primary antibodies used were listed in Supplementary Table 3.

### Luciferase reporter assay

Wild type and mutated TP53INP1 3'UTR was cloned to psiCHECK-2 Vector (Promega, Madison, WI, USA) to construct the psiCHECK-TP53INP1 3'UTR-wt and psiCHECK-TP53INP1 3'UTR-mut vectors with Lipofectamine 2000 Reagent (Invitrogen, Carlsbad, CA, USA). miR-96 sponge and its control hepatoma cells were transfected with TP53INP1 WT or TP53INP1 mutant 3'UTR plasmids for 48h. The luciferase activity was measured using a Synergy 2 Multidetection Microplate Reader (BioTek Instruments, Inc.). The data were normalized for transfection efficiency by dividing firefly luciferase activity by Renillaluciferase activity.

### Apoptosis assay

miR-96 mimic or miR-96 sponge and their control hepatoma cells were treated with sorafenib (10 μM) for 48 hours, followed by staining with Annexin V and 7-AAD for 15 minutes at 48C in the dark. Apoptotic cells were determined by an Annexin VFITC Apoptosis Detection Kit I (BD Pharmingen, San Diego, CA) and detected by flow cytometry according to the manufacturer's instructions.

### Patient-derived xenograft

For the patient-derived xenograft (PDX) model, primary tumor samples were obtained for xenograft establishment as described previously. The mice with xenografts were given sorafenib (60 mg/kg) or vehicle daily orally for 24 days (n=5 for each group). Tumor volumes were measured at the end time points. All procedures and protocols were approved by the Institutional Review Board of Eastern Hepatobiliary Surgery Hospital.

### Statistical analysis

GraphPad Prism (GraphPad Software, Inc. La Jolla, USA) was used for all statistical analyses. Statistical analysis was carried out using *t* test or Bonferroni Multiple Comparisons Test: *p<0.05. A *p* value of less than 0.05 was considered significant.

## Results

### The expression of miR-96 is upregulated in liver T-ICs

**Figure 1:**
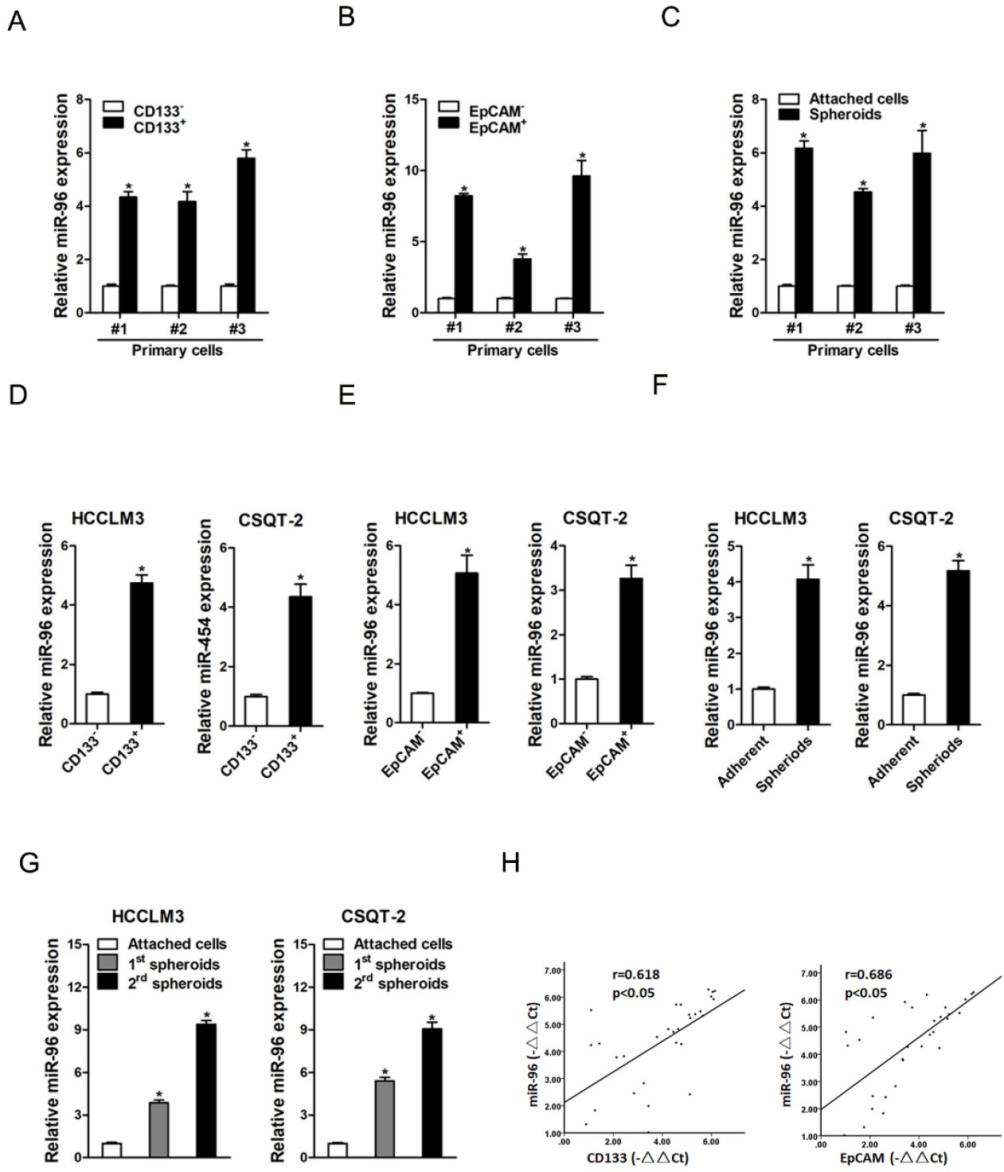
** miR-96 is upregulated in liver T-ICs. A.** The expression of miR-96 in MACS sorted CD133^+^ primary HCC cells was checked by real-time PCR assay (n=3). **B.** The expression of miR-96 in MACS sorted EpCAM^+^ primary HCC cells was checked by real-time PCR assay (n=3). **C.** The expression of miR-96 in primary HCC adherent and spheroids cells was checked by real-time PCR assay (n=3). **D.** Real-time PCR was performed to check the expression of miR-96 in MACS sorted CD133^+^ HCC cells (n=3). **E.** Real-time PCR was performed to check the expression of miR-96 in MACS sorted EpCAM^+^ HCC cells (n=3). **F.** Real-time PCR was performed to check the expression of miR-96 in HCC adherent and spheroids cells (n=3). **G.** miR-96 expression in serial passages of HCCLM3 and CSQT-2 spheroids was analyzed by real-time PCR (n=3). **H.** The correlation between the transcription level of miR-96 and CD133 or EpCAM in thirty HCC tissues was determined by real-time PCR analysis. Data were normalized to U6 or β-actin as △Ct and analyzed by Spearman's correlation analysis (data are represented as mean±s.d.; *P<0.05; two-tailed Student's *t*-test).

It is well known that CD133 and EpCAM are liver T-ICs markers 16, 17. We isolated CD133 and EpCAM positive liver T-ICs from primary HCC patients and HCC cell lines. As shown in Fig. 1A & B, miR-96 expression was upregulated in CD133^+^ and EpCAM^+^ liver T-ICs that were sorted from primary HCC patients. Compared with the attached cells, miR-96 expression was increased in HCC spheres derived from human primary HCC cells (Fig. 1C). Consistently, miR-96 expression was also upregulated in CD133^+^ and EpCAM^+^ liver T-ICs that were sorted from HCC cell lines (Fig. 1 D & E). Moreover, miR-96 expression was increased in the self-renewing spheroids compared with the attached cells (Fig. 1F). In serial passages of HCCLM3 or CSQT-2 spheroids, miR-96 expression was gradually increased (Fig. 1G). More importantly, in HCC tissues, pearson correlation analysis demonstrated that miR-96 levels were negatively correlated with the expression of CD133 and EpCAM (Fig. 1H). Taken together, these results demonstrated that miR-96 was preferentially upregulated in liver T-ICs.

### miR-96 depletion suppresses liver T-ICs expansion

**Figure 2:**
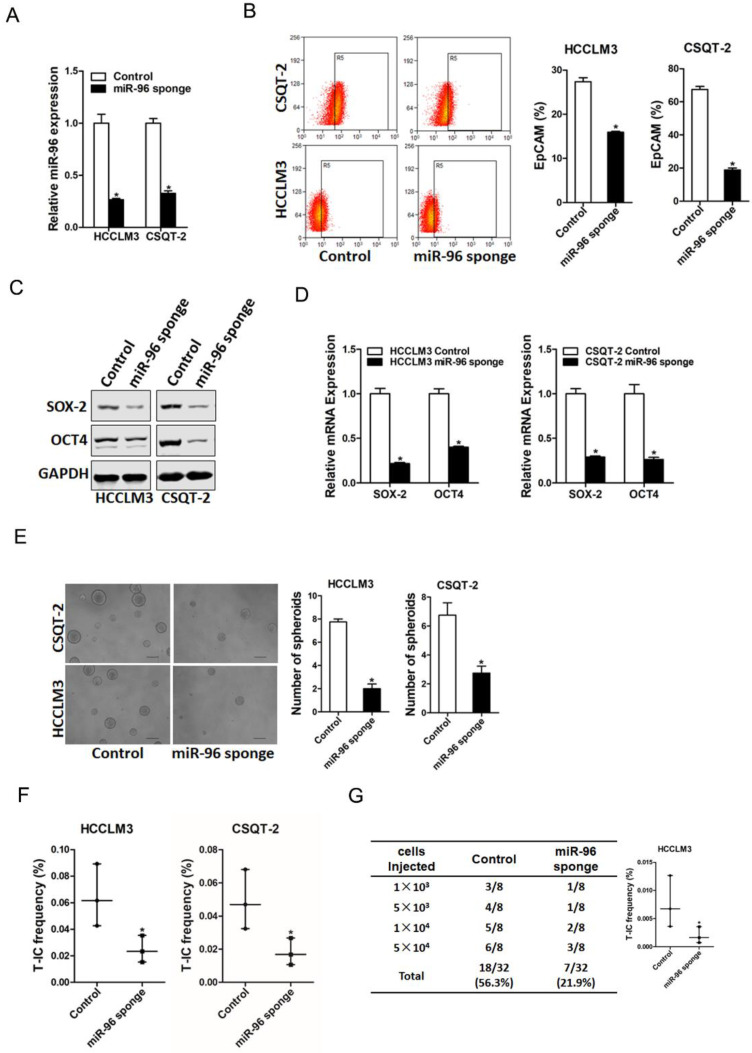
** miR-96 knockdown inhibits liver T-ICs expansion. A.** HCCLM3 and CSQT-2 cells were infected with miR-96 sponge virus and the stable infectants were determined by real-time PCR (n=3). **B.** The expression of liver T-ICs surface marker EpCAM miR-96 sponge and control hepatoma cells was checked by flow-cytometry analyses (n=3). **C.** The protein expression of SOX-2 and OCT4 was checked in miR-96 sponge and control hepatoma cells (n=3). **D.** The mRNA expression of SOX-2 and OCT4 was checked in miR-96 sponge and control hepatoma cells (n=3). **E.** Spheres formation assay of miR-96 sponge and control hepatoma cells (n=3). **F.** The frequency of liver T-ICs in miR-96 sponge and control hepatoma cells was compared by *in vitro* limiting dilution assay (n=16). **G.** The frequency of liver T-ICs in miR-96 sponge and control hepatoma cells was compared by *in vivo* limiting dilution assay (n=8). The frequency of tumor initiating cells was assessed using ELDA software (data are represented as mean±s.d.; *P<0.05; two-tailed Student's *t*-test).

To explore the biological function of miR-96 in liver T-ICs, HCCLM3 or CSQT-2 cells were infected with miR-96 sponge virus and the interference effect was confirmed by real-time PCR assay (Fig. 2A). Flow-cytometry analysis showed that EpCAM^+^ HCC cells was reduced in miR-96 knockdown hepatoma cells (Fig. 2B). Moreover, the expression of pluripotent transcription factors in miR-96 knockdown hepatoma cells was downregulated (Fig. 2C & D). Additionally, miR-96 interference hepatoma cells formed smaller and fewer spheroids than control cells (Fig. 2E). Furthermore, *in vitro* and *in vivo* limiting dilution assay revealed that suppression of miR-96 significantly reduced T-ICs frequency and tumorigenesis ability in hepatoma cells (Fig. 2F & G).

### miR-96 promotes liver T-ICs expansion

**Figure 3:**
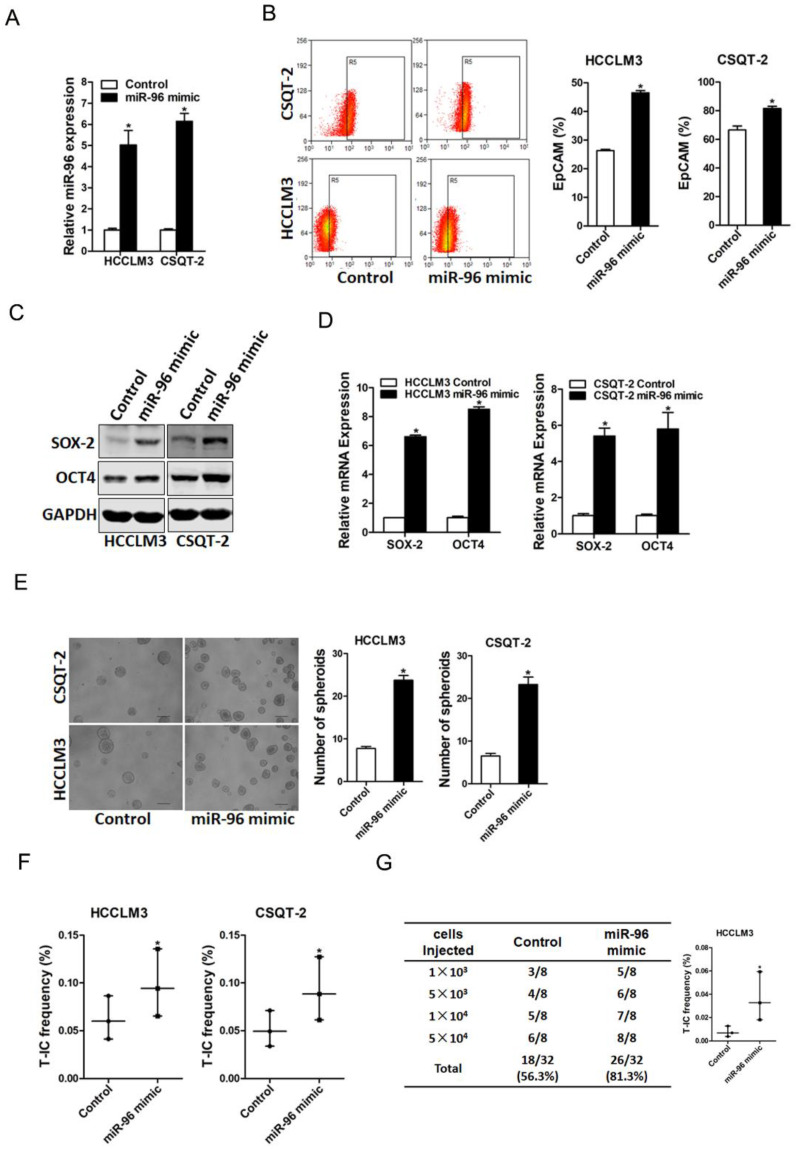
** miR-96 overexpression promotes the expansion of liver T-ICs. A.** HCCLM3 and CSQT-2 cells were infected with miR-96 mimic virus and the stable infectants were determined by real-time PCR (n=3). **B.** The expression of liver T-ICs surface marker EpCAM miR-96 mimic and control hepatoma cells was checked by flow-cytometry analyses (n=3). **C.** The protein expression of SOX-2 and OCT4 was checked in miR-96 mimic and control hepatoma cells (n=3). **D.** The mRNA expression of SOX-2 and OCT4 was checked in miR-96 mimic and control hepatoma cells (n=3). **E.** Spheres formation assay of miR-96 mimic and control hepatoma cells (n=3). **F.** The frequency of liver T-ICs in miR-96 mimic and control hepatoma cells was compared by *in vitro* limiting dilution assay (n=16). **G.** The frequency of liver T-ICs in miR-96 mimic and control hepatoma cells was compared by *in vivo* limiting dilution assay (n=8). The frequency of tumor initiating cells was assessed using ELDA software (data are represented as mean±s.d.; *P<0.05; two-tailed Student's *t*-test).

To further explore the role of miR-96 in liver T-ICs, HCCLM3 or CSQT-2 cells were infected with miR-96 mimic virus and the overexpress effect was confirmed by real-time PCR assay (Fig. 3A). Flow-cytometry analysis showed that EpCAM^+^ HCC cells were increased in miR-96 overexpressing hepatoma cells (Fig. 3B). Moreover, the expression of pluripotent transcription factors in miR-96 overexpressing hepatoma cells was upregulated (Fig. 3C & D). Additionally, miR-96 overexpressing hepatoma cells formed much more spheroids than control cells (Fig. 3E). Furthermore, *in vitro* and *in vivo* limiting dilution assay revealed that overexpression of miR-96 significantly increased T-ICs frequency and tumorigenesis ability in hepatoma cells (Fig. 3F & G).

### TP53INP1 is required for miR-96 mediated T-ICs expansion

**Figure 4:**
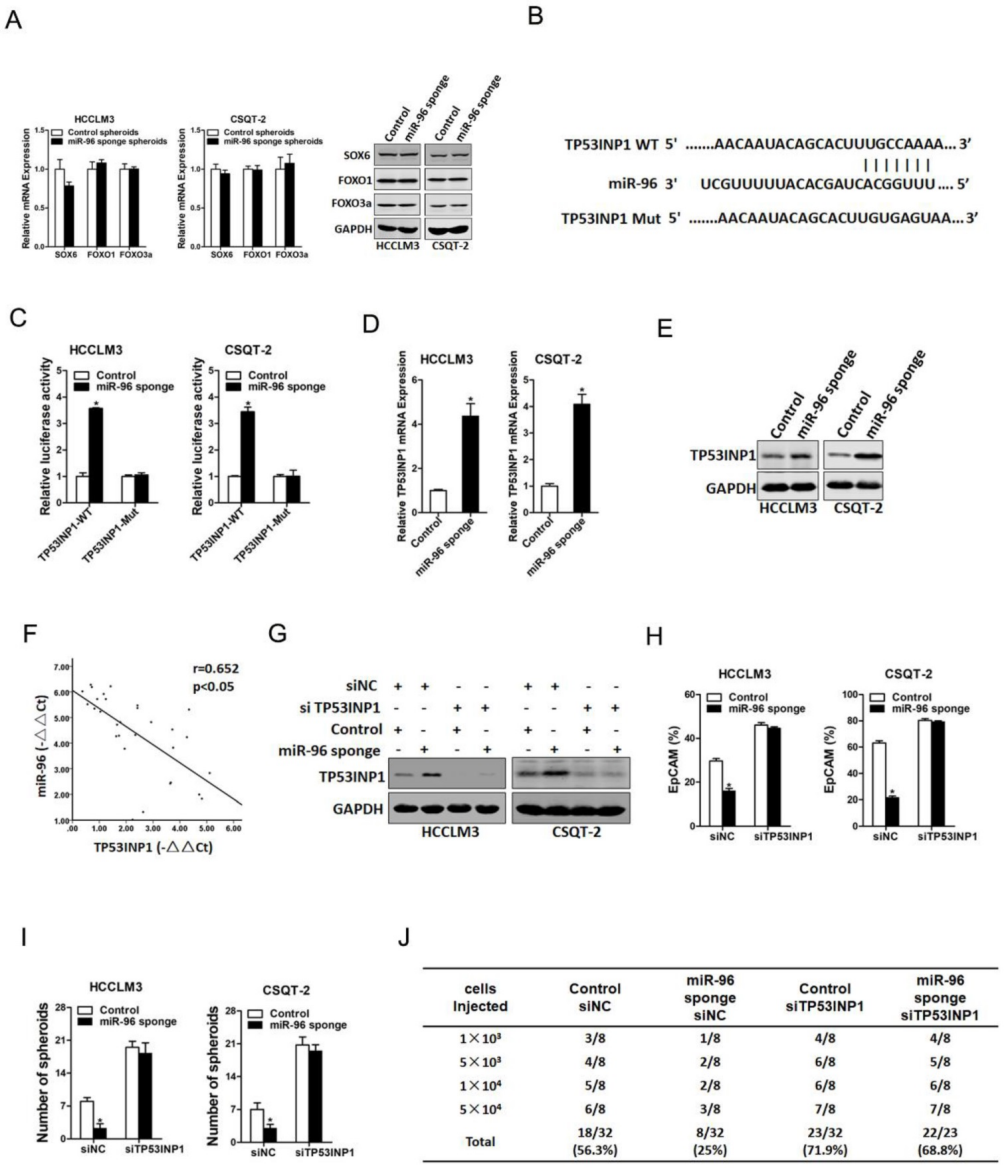
** TP53INP1 is a direct target of miR-96 in liver T-ICs. A.** The mRNA of SOX6, FOXO1 and FOXO3a in miR-96 sponge spheroids and control hepatoma spheroids was determined by real-time PCR and western blot assay (n=3). **B.** TargetScan of miR-96 potential binding sites at the 3'UTR of TP53INP1 and the nucleotides mutated in the TP53INP1-3'UTR mutant. **C.** Luciferase reporter assays performed in miR-96 sponge and control cells transfected with wild-type or mutant TP53INP1 3'-UTR constructs (n=3). **D.** The protein expression of TP53INP1 in miR-96 sponge spheroids and control hepatoma spheroids was checked by western blot assay (n=3). **E.** The protein expression of TP53INP1 in miR-96 sponge spheroids and control hepatoma spheroids was checked by western blot assay. **F.** Spearman correlation analysis of the relationship between TP53INP1 mRNA and miR-96 expression in 30 HCC specimens. **G.** miR-96 sponge and control hepatoma cells were transfected with TP53INP1 siRNA or control siRNA and subjected to western blot assay (n=3). **H.** miR-96 sponge and control hepatoma cells were transfected with TP53INP1 siRNA or control siRNA and the EpCAM^+^ hepatoma cells were checked by flow-cytometric assay (n=3). **I.** miR-96 sponge and control hepatoma cells were transfected with TP53INP1 siRNA or control siRNA and subjected to spheroid formation (n=3). **J.** miR-96 sponge and control hepatoma cells were transfected with TP53INP1 siRNA or control siRNA and were then injected subcutaneously into NOD-SCID mice. Tumors incidence was observed over 2 months; n=8 for each group (data are represented as mean±s.d.; *P<0.05; two-tailed Student's *t*-test).

It was reported that miR-96 targeted the 3ʹ-UTR of SOX6, FOXO1 and FOXO3a in hepatoma cells (18, 19). So, we doubted whether SOX6, FOXO1 and FOXO3a were involved in miR-96 mediated liver T-ICs expansion. Our data found that SOX6, FOXO1 and FOXO3a expression was unchanged in miR-96 knockdown liver T-ICs (Fig. 4A). Next, we used TargetScan to predict the direct targets and found that TP53INP1 harbored potential miR-96 binding site (Fig. 4B). To further explore whether miR-96 directly regulates TP53INP1 expression via interaction with its 3'-UTR, the wild-type or mutant TP53INP1 3'-UTR reporter plasmids were transfected into miR-96 interference liver T-ICs. The mutation of miR-96 binding site in the TP53INP1 3'-UTR diminished the distinct activation of TP53INP1 3'-UTR between miR-96 knockdown liver T-ICs and control cells (Fig. 4C). Moreover, we found that TP53INP1 mRNA and protein expression was upregulated in miR-96 knockdown liver T-ICs (Fig. 4D & E). Consistently, there was a significant negative correlation between miR-96 and TP53INP1 mRNA expression in HCC samples (Fig. 4F).

Next, we explore the expression of TP53INP1 in liver T-ICs. As shown in Supplementary Fig. 1A & B, TP53INP1 expression was reduced in CD133^+^ and EpCAM^+^ liver T-ICs that were sorted from primary HCC patients. Compared with the attached cells, TP53INP1 expression was downregulated in HCC spheres derived from human primary HCC cells (Supplementary Fig. 1C). Moreover, TP53INP1 expression was decreased in CD133^+^ and EpCAM^+^ liver T-ICs that were sorted from HCC cell lines (Supplementary Fig. 1D & E). Consistently, we also found that TP53INP1 expression was downregulated in HCC spheres derived from human HCC cell lines (Supplementary Fig. 1F). To further confirm the role of TP53INP1 in miR-96-mediated liver T-ICs expansion, the TP53INP1 siRNA was used (Fig. 4G). TP53INP1 siRNA diminished the discrepancy of expression of T-ICs markers, self-renewal ability, and tumorigenesis capacity between miR-96 knockdown hepatoma cells and control cells (Figure 4H-J).

### miR-96 overexpression HCC cells are resistant to sorafenib treatment

**Figure 5:**
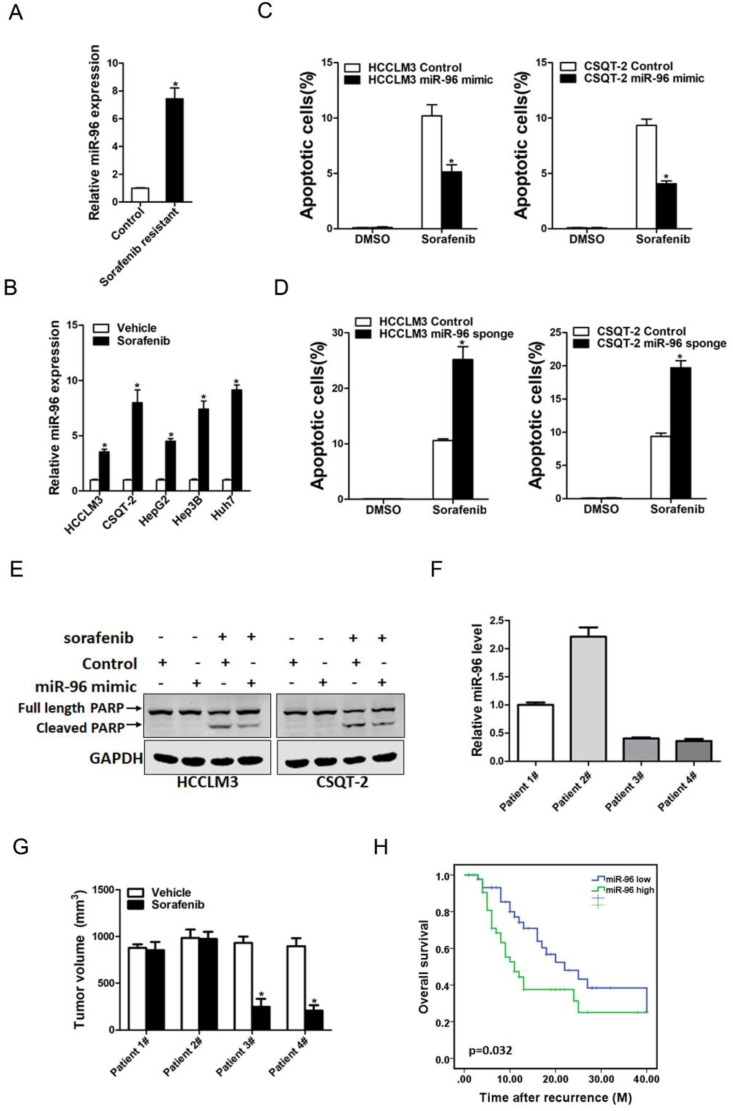
** miR-96 is associated with the sensitivity of sorafenib. A.** The expression of miR-96 in sorafenib resistant PDXs were checked by real-time PCR assay (n=6). **B.** The expression of miR-96 in sorafenib resistant HCC cell lines were checked by real-time PCR assay (n=3). **C.** miR-96 mimic and control hepatoma cells were treated with sorafenib (10 µM) for 48 hours and their apoptosis was checked by flow cytometry (n=3). **D.** miR-96 sponge and control hepatoma cells were treated with sorafenib(10 µM) for 48 hours and their apoptosis was checked by flow cytometry (n=3). **E.** miR-96 mimic and control hepatoma cells were treated with 10 µM sorafenib as indicated for 48 hours. The protein of cleaved-PARP was determined by western blot. F. The expression of miR-96 in PDXs primary tumors was determined by RT-PCR assay (n=4). **G.** PDXs with low or high miR-96 expression in their primary tumors were treated with sorafenib (60 mg/kg body weight) or vehicle for 30 days (n=6 for each group). The terminal tumor size and weight was showed. **H.** The overall survival of patients between miR-96-high (n=46) or miR-96-low (n=45) groups was evaluated by Kaplan-Meier analysis in HCC patients who received sorafenib after recurrence (data are represented as mean±s.d.; *P<0.05; two-tailed Student's *t*-test).

Increasing evidence shows that liver T-ICs were closely associated with HCC chemoresistance. Thus, we next explored the correlation between miR-96 expression and sorafenib response in HCC patients. miR-96 expression was significantly upregulated in sorafenib-resistant HCC xenografts (Fig. 5A). Consistently, we also found that miR-96 expression was increasing in sorafenib-resistant hepatoma cells (Fig. 5B). miR-96 overexpression led to the resistance of hepatoma cells upon sorafenib-induced cell apoptosis (Fig. 5C). Moreover, miR-96 knockdown sensitized hepatoma cells to sorafenib-induced cell apoptosis (Fig. 5D). In addition, western blot analysis also showed that the protein expression of cleaved-PARP in miR-96 overexpressing hepatoma cells was decreased when they were exposed to the same doses of sorafenib (Fig. 5E). Conversely, western blot analysis showed that the protein expression of cleaved-PARP in miR-96 knockdown hepatoma cells was increased when they were exposed to the same doses of sorafenib (Supplementary Fig. 2A). Furthermore, we found that the PDXs derived from HCC tumors with high miR-96 levels were resistant to sorafenib treatment. In contrast, sorafenib eliminated the growth of PDXs derived from the HCC tumors with low miR-96 levels compared with the vehicle controls (Fig. 5F & G). More importantly, Kaplan-Meier analysis indicated that low miR-96 levels in the primary HCCs were significantly associated with prolonged overall survival in patients received sorafenib to treat their recurrent tumors (Fig. 5H), which further demonstrating that the miR-96 expression in HCC patients can serve as a reliable predictor for sorafenib response.

## Discussion

Most cancer therapies fail to eradicate tumors due to the existence of T-ICs. However, the understanding of regulatory mechanisms for T-ICs is limited. In the present study, we demonstrated the critical role of miR-96 in liver T-ICs and the underlying mechanism. To our knowledge, this is the first report for miR-96 in the regulation of liver T-ICs.

The existence of T-ICs has been confirmed by numerous studies, and these cells have the ability to self-renew and the potential for generating heterogeneous malignant progenies 20, 21. CD133 or EpCAM are well-known liver T-ICs markers. We noted that that miR-96 levels increased in CD133^+^ or EpCAM^+^ primary liver T-ICs. Spheroid culture of cancer cells is a routine approach to enrich T-ICs. We also found that miR-96 expression was upregulated in hepatoma spheroids. It was well-accepted that liver T-ICs were closely associated with the chemoresistance and HCC recurrence. Interestingly, the expression of miR-96 in these sorafenib-resistant xenografts was dramatically increased.

Accumulating evidence shows that miRNAs are involved in the regulation of human cancers and can be used as the diagnosis and therapeutic targets 22, 23. For instance, miR-96 is upregulated in breast cancer and promotes breast cancer metastasis by suppressing MTSS1 24. It was also reported that miR-96 works as a tumor repressor by inhibiting NPTX2 in renal cell carcinoma 25. However, the potential function of miR-96 in liver T-ICs has not been reported. In the present study, we found that miR-96 expression is significantly upregulated in CD133^+^ or EpCAM^+^ primary liver T-ICs. Moreover, miR-96 can promote the self-renewal capacity and tumorigenicity of liver T-ICs.

It was widely recognized that TP53INP1 gene is an important tumor suppressor. TP53INP1 suppressed number cancers initiation and progression 26, 27. Previous studies have elucidated that TP53INP1plays essential roles in HCC. For instance, TP53INP1 was downregulated in HCC tissues and regulated the metastasis of HCC cells 28. Exosomal miR-93 promotes proliferation and invasion in hepatocellular carcinoma by directly inhibiting TIMP2/TP53INP1/CDKN1A 29. In addition, TP53INP1 was reported to be involved in the regulation of cancer stemness 30, 31. We hereby revealed that knockdown miR-96 upregulated TP53INP1 mRNA and protein expression in liver T-ICs. Moreover, we found that miR-96 directly regulates TP53INP1 expression via interaction with its 3'-UTR. TP53INP1 siRNA further confirm that miR-96 via TP53INP1 pathway promotes liver T-ICs expansion.

Sorafenib is the first FDA-approved targeted drug which was used for the treatment of advanced HCC patients 32, 33. However, only a small part of HCC patients is benefited from sorafenib treatment. Therefore, it is important to find the right population of patients for sorafenib treatment. In this study, our finding revealed that miR-96 knockdown HCC cells are more sensitive to sorafenib treatment. The analysis of patient cohorts and PDX studies further confirmed that low miR-96 level in HCC patients can serve as a reliable predictor for sorafenib response.

In conclusion, we demonstrated for the first time that miR-96 expression is upregulated in liver T-ICs, and miR-96 shRNA silencing suppresses the self-renewal and tumorigenesis of liver T-ICs. Moreover, miR-96 promoted liver T-ICs expansion by directly targeting TP53INP1. The findings of the present study not only shed new light on the mechanisms responsible for liver T-ICs expansion but also suggest that miR-96 may be a potential therapeutic target for liver T-ICs.

## Supplementary Material

Supplementary figures and tables.

## Abbreviations

T-ICs: tumor-initiating cells
CSCs: cancer stem cells
miRNAs: MicroRNAs
HCC: hepatocellular carcinoma
3'-UTR: 3'-untranslational region
DMEM: Dulbecco's modified Eagle's medium
FBS: fetal bovine serum
APC: adenomatous polyposis coli
PDXs: patient-derived xenografts
