# Supplementary material for: miR-96 regulates liver tumor-initiating cells expansion by targeting TP53INP1 and predicts Sorafenib resistance
Source: J Cancer. 2020 Sep 21;11(22):6545–55. doi: 10.7150/jca.48333 (PMC7545665; doi:10.7150/jca.48333)
Supplement: Supplementary file 1 — Supplementary figures and tables. [file jcav11p6545s1.pdf]

miR-96 Regulates Liver Tumor-Initiating Cells Expansion by targeting TP53INP1 and Predicts Sorafenib Resistance

Yonggang Huang[1,#], Jin Zhang[2,#], HengYu Li[3,#], Huiping Peng[4,*], Maolin Gu[1,*], Hengjie Wang[1,*]

[1]Department of Hepatic surgery, Kunshan Hospital of Traditional Chinese Medicine. Kunshan, Jiangsu Province, 215300, China;
[2]Department of Hepatic Surgery, Third Affiliated Hospital of Second Military Medical University, Shanghai, 200438, China;
[2]Department of General surgery, First Affiliated Hospital of Second Military Medical University, Shanghai, 200433, China;
[4]Department of Gastroenterology, Kunshan Hospital of Traditional Chinese Medicine. Kunshan, Jiangsu Province, 215300, China.
[#]These authors contributed equally to this work
Correspondence author address: Department of Hepatic Surgery, Kunshan of traditional Chinese hospital, Kunshan, Jiangsu Province, 215300, China. E-mail addresses: php751129@sina.com (Huiping Peng), gmlkunshan@163.com (Maolin Gu) and xiaowang198587@163.com (Hengjie Wang).

Running title: miR-96 promotes liver T-ICs expansion.

Funding:
This work was supported by grants from the National Natural Science Foundation of China (81572791, 81101578), Kunshan science and technology development project (KS19045), the National Natural Science Foundation of Shanghai (18ZR1438600). The funding was used in the design of the study and collection, analysis, and interpretation of data and in writing the manuscript.

Supplementary Figure 1

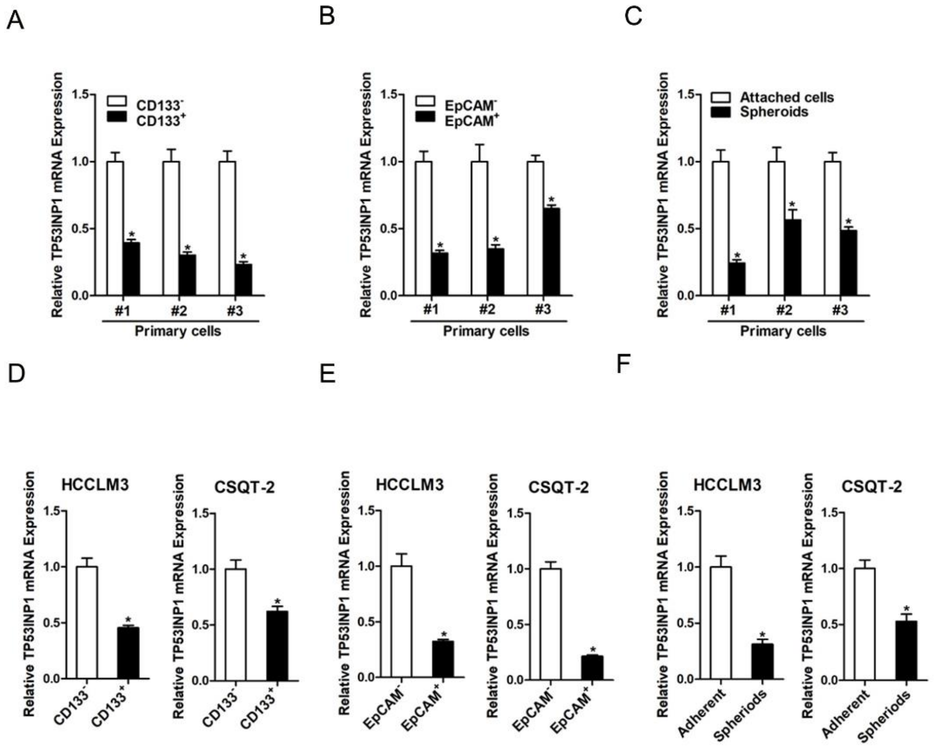

Supplementary Figure 1.

A. The expression of TP53INP1 in $CD133^{+}$ and $CD133^{-}$ primary HCC cells was checked by real-time PCR assay. (n=3)

B. The expression of TP53INP1 in $EpCAM^{+}$ and $EpCAM^{-}$ primary HCC cells was checked by real-time PCR assay. (n=3)

C. Realtime–PCR analysis of TP53INP1 in primary HCC adherent and spheroids cells. (n=3)

D. The expression of TP53INP1 in $CD133^{+}$ and $CD133^{-}$ HCC cell lines was checked by real-time PCR assay. (n=3)

E. The expression of TP53INP1 in $EpCAM^{+}$ and $EpCAM^{-}$ HCC cell lines was checked by real-time PCR assay. (n=3)

F. Realtime–PCR analysis of TP53INP1 in human HCC adherent and spheroids cells. (n=3)

(Data are represented as mean±s.d.; *P＜0.05; two-tailed Student's t-test.)

Supplementary Figure 2

A

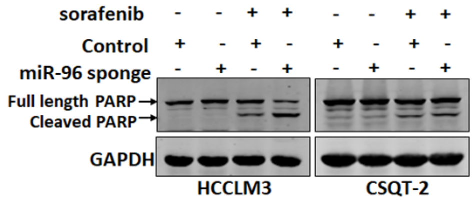

Supplementary Figure 1.

A. miR-96 knockdown and control hepatoma cells were treated with 10 μM sorafenib as indicated for 48 hours. The protein of cleaved-PARP was determined by western blot.

Supplementary Table 1. Clinicopathologic Features of 91 HCC Specimens

Characteristics		miR-96 low (n=46)	miR-96 high (n=45)	p value
Age(year)	≤50	24	25	>0.05
	>50	22	21	
Gender	Male	38	37	>0.05
	Female	8	8	
HBsAg	Positive	29	42	<0.05
	Negative	17	3	
AFP(μg/L)	≤400	26	27	>0.05
	>400	20	18	
Tumor size(cm)	≤5	21	19	>0.05
	>5	25	26	
Tumor number	Single	38	27	<0.05
	Multiple	8	18	
Portal vein tumor thrombus	Yes	6	4	>0.05
	No	40	41	
Pathological satellite	Yes	21	32	<0.05
	No	25	13	
BCLC stage	A	32	13	
	B or C	14	32	<0.05

HBsAg: hepatitis B virus surface antigen; AFP: α-fetoprotein; BCLC: Barcelona Clinic Liver Cancer Staging.

Supplementary Table 2. Primer List.

Gene	Forward primer	Reverse primer (5'-3')
β-actin(Human)	Forward (5′- 3′)	GGCCCAGAATGCAGTTCGCCTT
	Reverse (5′- 3′)	AATGGCACCCTGCTCACGCA
SOX2(Human)	Forward (5′- 3′)	TGGAGAAGGAATGGTCCACTTC
	Reverse (5′- 3′)	GGATAAGTACACGCTGCCCG
OCT4(Human)	Forward (5′- 3′)	ATGTGCGCGTAACTGTCCAT
	Reverse (5′- 3′)	CTGCAGTGTGGGTTTCGGGCA
SOX6(Human)	Forward (5′- 3′)	CCTCTACCTCACCACATAAGC
	Reverse (5′- 3′)	TCCACCACATCGGCAAGA
FOXO1(Human)	Forward (5′- 3′)	GACAGCCCTGGATCACAGTTT
	Reverse (5′- 3′)	CGGTCATAATGGGTGAGAGTCT
FOXOa3(Human)	Forward (5′- 3′)	TTCCGTAAGCAAGCCGTGTA
	Reverse (5′- 3′)	CGAGTCCGAAGTGAGCAGGT

TP53INP1 (Human)	Forward (5′- 3′)	CCCCACCCCCATGTTTTACT
	Reverse (5′- 3′)	TTTCCTGGCCCTGGGACTAC
miR-96 (Human)	Forward (5′- 3′)	AGCAAAAATGTGCTAGTGCCAAA
	Reverse (5′- 3′)	AGCAAAAATGTGCTAGTGCCAAA
U6 (Human)	Forward (5′- 3′)	CTCGCTTCGGCAGCACA
	Reverse (5′- 3′)	AACGCTTCACGAATTTGCGT

Supplementary Table 3. Antibody List.

**Antigens**	**Manufacturer**	**Application**
TP53INP1	Abcam, Cambridge, MA	1:500 for WB
PARP	Cell Signaling Technology, Beverly, MA	1:500 for WB
GAPDH	Cell Signaling Technology, Beverly, MA	1:5000 for WB
SOX-2	Cell Signaling Technology, Beverly, MA	1:500 for WB
OCT4	Cell Signaling Technology, Beverly, MA	1:500 for WB
